# Phosphoproteomic analysis reveals changes in A-Raf-related protein phosphorylation in response to *Toxoplasma gondii* infection in porcine macrophages

**DOI:** 10.1186/s13071-024-06273-x

**Published:** 2024-04-20

**Authors:** Dingzeyang Su, Shifan Zhu, Kangzhi Xu, Zhaofeng Hou, Fuxing Hao, Fan Xu, Yifan Lin, Yuyang Zhu, Dandan Liu, Qiangde Duan, Xinjun Zhang, Yuguo Yuan, Jinjun Xu, Jianping Tao

**Affiliations:** 1 College of Veterinary Medicine, Yangzhou University, 12 East Wenhui Road, Yangzhou, Jiangsu 225009 People’s Republic of China; 2 Jiangsu Co-Innovation Center for Prevention and Control of Important Animal Infectious Diseases and Zoonosis, Yangzhou, 225009 People’s Republic of China; 3 International Research Laboratory of Prevention and Control of Important Animal Infectious Diseases and Zoonotic Diseases of Jiangsu Higher Education Institutions, Yangzhou University, Yangzhou, 225009 People’s Republic of China; 4 Jiangsu Agri-Animal Husbandry Vocational College, Taizhou, 225300 People’s Republic of China

**Keywords:** *Toxoplasma gondii*, Host cells, Apoptosis, Phosphorylation, A-Raf, 3D4/21

## Abstract

**Background:**

*Toxoplasma gondii* is an obligate intracellular protozoan parasite that causes severe threats to humans and livestock. Macrophages are the cell type preferentially infected by *T. gondii *in vivo. Protein phosphorylation is an important posttranslational modification involved in diverse cellular functions. A rapidly accelerated fibrosarcoma kinase (A-Raf) is a member of the Raf family of serine/threonine protein kinases that is necessary for MAPK activation. Our previous research found that knockout of A-Raf could reduce *T. gondii*-induced apoptosis in porcine alveolar macrophages (3D4/21 cells). However, limited information is available on protein phosphorylation variations and the role of A-Raf in macrophages infected with *T. gondii*.

**Methods:**

We used immobilized metal affinity chromatography (IMAC) in combination with liquid chromatography tandem mass spectrometry (LC–MS/MS) to profile changes in phosphorylation in *T. gondii-*infected 3D4/21 and 3D4/21-Δ*Araf* cells.

**Results:**

A total of 1647 differentially expressed phosphorylated proteins (DEPPs) with 3876 differentially phosphorylated sites (DPSs) were identified in *T. gondii*-infected 3D4/21 cells (p3T group) when compared with uninfected 3D4/21 cells (pho3 group), and 959 DEPPs with 1540 DPSs were identified in the p3T group compared with infected 3D4/21-Δ*Araf* cells (p3KT group). Venn analysis revealed 552 DPSs corresponding to 406 DEPPs with the same phosphorylated sites when comparing p3T/pho3 versus p3T/p3KT, which were identified as DPSs and DEPPs that were directly or indirectly related to A-Raf.

**Conclusions:**

Our results revealed distinct responses of macrophages to *T. gondii* infection and the potential roles of A-Raf in fighting infection via phosphorylation of crucial proteins.

**Graphical Abstract:**

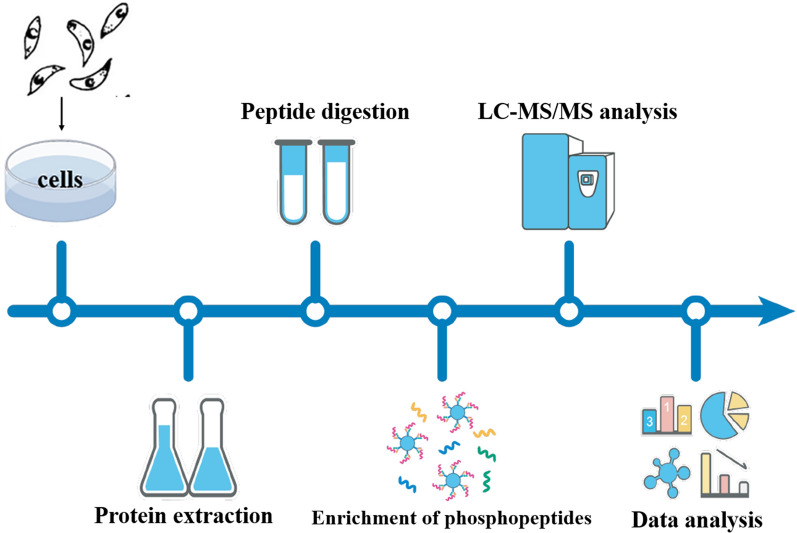

**Supplementary Information:**

The online version contains supplementary material available at 10.1186/s13071-024-06273-x.

## Background

*Toxoplasma gondii* is an obligate intracellular parasite that can infect almost all warm-blooded animals, including humans [1]. Approximately one-third of humans worldwide are infected with *T. gondii* [2]. Human infections are mainly acquired via ingestion of undercooked meat containing cysts or water and fruit contaminated with sporulated oocysts [3]. The majority of infections seem to show no symptoms in individuals with a healthy immune system [4]. However, *T. gondii* infections can induce abortion or fetal developmental disorders in pregnant women [5] and cause serious health problems and even death in immunocompromised individuals [6]. In the field of animal husbandry, sheep, goats, and pigs are highly vulnerable to *T. gondii* infection, resulting in significant economic losses worldwide [7]. Therefore, toxoplasmosis is of great importance in both veterinary and human medicine. However, the existing approaches to address toxoplasmosis in humans and animals are considerably inadequate [8].

Protein posttranslational modification (PTM), a crucial regulator of protein function, is necessary for the structure and operation of proteins under both normal and abnormal circumstances [9]. Protein phosphorylation is a PTM process [10] that has been shown to affect almost every cellular process [11]. In eukaryotes, approximately 30% of all proteins can undergo phosphorylation. To date, thousands of different phosphorylation sites have been discovered, with phosphorylation on serine (S), threonine (T), and tyrosine (Y) being most common [12]. *T. gondii* infection can lead to changes in phosphorylation modifications of host proteins. For example, *T. gondii* infection induces phosphorylation of the Bad protein in host cells, which regulates the PI3K-PKB/AKT signaling pathway and inhibits apoptosis of host cells [13]. During the initial phases of invasion, ROP16, a protein kinase from *T. gondii*, can activate and phosphorylate transcription factors associated with the immune response, such as signal transducer and activator of transcription (STAT) 3/5/6, and can also influence host signaling pathways in varies ways depending on the strain [14–16]. The molecules involved in phosphorylation modification may be new targets for the control of toxoplasmosis.

A-rapidly accelerated fibrosarcoma (A-Raf) is a serine/threonine protein kinase and a member of the Raf family of protein kinases, which consists of the A-Raf, B-Raf, and C-Raf isoforms [17], and regulates a variety of basic cellular functions, including proliferation, differentiation, transformation, apoptosis, and metabolism, mainly through the MEK/ERK pathway [18]. In cells lacking B-Raf and C-Raf, A-Raf can directly phosphorylate MEK1 [17]. Previous studies revealed that A-Raf can regulate apoptosis by inhibiting MST2 phosphorylation [19] and antagonize node/Smad2 signaling by directly phosphorylating the Smad2 junctional region and attenuating Smad2 signaling [20]. A-Raf has also been shown to be associated with phosphorylation of the platelet-derived growth factor receptor (PDGFR) [21]. In our previous study, we observed that *T. gondii* infection inhibits expression of A-Raf in porcine alveolar macrophages (3D4/21 cells), and knockout of the *Araf* gene led to decreased apoptosis of host cells induced by *T. gondii* infection [22]. However, the changes in phosphorylation modifications of porcine macrophages induced by *T. gondii* infection and the roles of A-Raf in regulating the apoptosis of porcine macrophages via phosphorylation modifications were unclear.

In the present study, we conducted a phosphoproteomic analysis of both 3D4/21 cells and 3D4/21-Δ*Araf* cells (*Araf* gene knockout) infected with the *T. gondii* Chinese I genotype (ToxoDB #9) strain. The aim was to identify phosphorylated proteins and sites potentially regulated by A-Raf. Our data provide new insight into the role of phosphorylation modifications in *T. gondii* infection, which may pave the way for exploring new targets for the prevention and control of toxoplasmosis as well as for the understanding of *Toxoplasma*–pig interactions.

## Methods

### Parasites and cells

The *T. gondii* YZ-1 strain, isolated from a home-bred wild boar that succumbed to illness on a private farm in Jiangsu Province, China, was identified as ToxoDB (Chinese I), which is the most prevalent genotype in China and was shown to be virulent in mice in our previous study [23]. The 3D4/21 cells (porcine alveolar macrophages) were purchased from Shanghai Zhong Qiao Xin Zhou Biotechnology Co., Ltd. (Shanghai, China). The cells were cultured at 37 °C with 5% CO_2_ in RPMI 1640 medium (Gibco, Shanghai, China) with 10% fetal bovine serum (FBS; Eallbio, Beijing, China) containing 100 IU/ml penicillin and 100 μg/ml streptomycin (Beyotime, Shanghai, China). The 3D4/21-Δ*Araf* cell line, a stable A-Raf gene knockout, was established in our previous study, and the cells were cultured as described previously [22].

### Infection and sample collection

The 3D4/21 cells and 3D4/21-Δ*Araf* cells were infected with *T. gondii* tachyzoites at a macrophage-to-tachyzoite ratio of 1:5 (multiplicity of infection of 5) or mock infected with an equal amount of phosphate-buffered saline (PBS, pH 7.4) [herein referred to as p3T (3D4/21 + *T. gondii*), p3KT (3D4/21-Δ*Araf* + *T. gondii*), and pho3 (3D4/21 + PBS)], respectively. After 24 h postinfection (PI), all samples were harvested and stored at −80 °C for subsequent protein extraction. All samples were confirmed to have no less than 2 × 10^7^ cells. Three independent biological replicates were performed for all experiments.

### Protein extraction and analysis

Proteins were extracted from pho3, p3T, and p3KT as follows. Samples were sonicated three times on ice using a high intensity ultrasonic processor (Scientz, Ningbo, China) in lysis buffer with 8 M urea, 1% protease inhibitor, and 1% phosphatase inhibitor cocktail (Beyotime, Shanghai, China). The supernatant containing total soluble proteins was collected after 10 min of centrifugation at 12,000*g* at 4 °C. After the total protein concentration was determined using a Bradford protein assay kit (Beyotime, Shanghai, China) according to the manufacturer’s instructions, 20 μg of each sample was separated by 12% sodium dodecyl sulfate polyacrylamide gel electrophoresis (SDS–PAGE) and transferred to nitrocellulose membranes (PALL, New York, USA). The membranes were blocked with 5% bovine serum albumin for 2 h at room temperature, then incubated with primary antiphosphothreonine antibody (1:500 dilution; PTM Bio, Hangzhou, China) overnight at 4 °C. The membranes were washed and incubated in horseradish peroxidase-conjugated goat anti-rabbit IgG (H + L) antibody (1:10,000 dilution; ABclonal, Wuhan, China) for 1 h at room temperature.

### Peptide digestion

An equal quantity of 1.1 mg of protein from each sample was prepared. Three technical replicates were analyzed for each biological replicate. For protein digestion, the volume was adjusted to consistency using lysate solution, a final concentration of 20% trichloroacetic acid was added slowly, vortexed and mixed, and precipitated at 4 °C for 2 h. The supernatant was discarded after centrifugation at 4500*g* for 5 min. The precipitate was washed with precooled acetone two to three times. After drying the precipitate, a final concentration of 200 mM triethylammonium hydrogen carbonate buffer was added, and the precipitate was broken up by ultrasonication. Trypsin was then added at a ratio of 1:50 (protease:protein, m/m) and digested overnight. Finally, the protein solution was reduced with 5 mM dithiothreitol for 30 min at 56 °C and incubated with 11 mM iodoacetamide for 15 min at room temperature in darkness.

### Enrichment of phosphopeptides using immobilized metal affinity chromatography (IMAC)

After peptide digestion, the resulting peptides were first incubated with an immobilized metal affinity chromatography (IMAC) microsphere suspension with vibration in loading buffer (50% acetonitrile/0.5% acetic acid). To remove nonspecifically adsorbed peptides, the IMAC microspheres were washed with buffer solution (50% acetonitrile/0.5% acetic acid) initially and then washed with buffer solution (30% acetonitrile/0.1% trifluoroacetic acid). To elute the enriched phosphopeptides, elution buffer containing 10% NH_4_OH was added, and the enriched phosphopeptides were eluted with vibration. For liquid chromatography tandem mass spectrometry (LC–MS/MS) analysis, the resulting peptides were desalted with C18 ZipTips (Millipore, Boston, USA) according to the manufacturer’s instructions.

### Liquid chromatography tandem mass spectrometry (LC–MS/MS) analysis

Enriched peptides were dissolved in liquid chromatography mobile phase A and separated using an EASY-nLC 1200 system (ThermoFisher Scientific, Waltham, USA). Mobile phase A was an aqueous solution containing 0.1% formic acid and 2% acetonitrile; mobile phase B was an aqueous solution containing 0.1% formic acid and 90% acetonitrile. The liquid phase gradient settings were as follows: 0–70 min, 3–18% phase B; 70–82 min, 18–28% phase B; 82–86 min, 28–80% phase B; and 86–90 min, 80% phase B, at a flow rate maintained at 500 nL/min. The peptides were separated using an ultrahigh-performance liquid chromatography (UHPLC) system and injected into the nanospray ion source (NSI) for ionization. The peptides were then analyzed by mass spectrometry on an Orbitrap Exploris™ 480 (ThermoFisher Scientific, Waltham, USA). The ion source voltage was set to 2.2 kV and the high-field asymmetric waveform ion mobility spectrometry (FAIMS) compensation voltage (CV) was set to −65 V, −45 V. The peptide parent ions and their secondary fragments were detected and analyzed using a high resolution Orbitrap. The primary mass spectrometry scan range was set to 400–1200 *m*/*z*, and the scan resolution was set to 60,000. The secondary mass spectrometry scan range was set to a fixed starting point of 110 *m*/*z*, the secondary scan resolution was set to 30,000, and TurboTMT was set to “off.” The data acquisition mode used a data-dependent scanning (DDA) procedure, i.e., the top 15 peptide ions with the highest signal intensity were selected after the primary scan. The peptide parent ions were sequentially fragmented into the high-energy collisional dissociation (HCD) collision cell using a fragmentation energy of 27%, again sequentially for secondary mass spectrometry. To improve the effective utilization of the mass spectra, the automatic gain control (AGC) was set to 75%, the signal threshold was set to 2.0 × 10^4^ ions/s, the maximum injection time was set to 100 ms, and the dynamic exclusion time of the tandem mass spectrometry scan was set to 30 s to avoid repeated scanning of the parent ions.

### Data analysis

The secondary mass spectrometry data were retrieved using PD2.4 (v2.4.1.15) for this experiment. The search parameters were set as follows: the database was Sus_scrofa_9823_PR_20220915.fasta (49,790 sequences), an inverse library was added to calculate the false positive rate (FDR) due to random matches, and a common contamination library was added to the database to remove the effect of contaminating proteins from the identification results. The digestion method was set to trypsin, carbamidomethyl (C) was set as a fixed modification, and oxidation (M), acetyl (N-terminus), met-loss (M), met-loss + acetyl (M), and phospho (S, T, Y) were set as variable modifications. The false discovery rate (FDR) values for protein, peptide, and PSM (peptide spectrum match) identification were all set to 1%.

### Phosphorylation motif analysis

The MoMo analysis tool, based on the motif-x algorithm, was used to characterize the motifs of the modification sites. Peptide sequences consisting of six amino acids upstream and downstream of all identified modification sites were used as the target of analysis. The background of analysis was peptide sequences consisting of six amino acids upstream and downstream of all potentially occurring modification sites in the species. When the number of peptides in a characteristic sequence form is greater than 20 and the *P* value of the statistical test is less than 1 × 10^–6^, the characteristic sequence form is a motif of the modified peptide [24].

### Bioinformatics analysis

The samples to be compared were selected, and the ratio of the mean relative quantitative values of the modification sites of multiple replicates was used as the fold of difference (fold change; FC). At *P* < 0.05, FC > 2 was considered significantly upregulated and at *P* < 0.05, FC < 0.5 was considered significantly downregulated. Gene ontology (GO) annotation was performed using eggnog-mapper software (v2.1.6) based on the EggNOG database [25]. Kyoto Encyclopedia of Genes and Genomes (KEGG) annotation and enrichment analysis were obtained from the KEGG pathway database (https://www.genome.jp/kegg/kegg3a.html) [26].

GO annotations of proteins are divided into three broad categories: biological process (BP), cellular component (CC), and molecular function (MF). KEGG pathway annotations of proteins are divided into five broad categories: cellular processes, environmental information processing, genetic information processing, metabolism, and organismal systems. Fisher’s exact tests were used to analyze the significance of annotations of differentially expressed proteins (using the identified protein as the background), and *P* < 0.05 was considered significant. The KEGG database was used for KEGG pathway enrichment analysis. Fisher’s exact tests were used to analyze the significance of KEGG pathway enrichment of differentially expressed proteins (using the identified protein as the background), and *P* < 0.05 was considered significant [27].

Venn analysis (https://bioinformatics.psb.ugent.be/webtools/Venn/) was used to determine and compare the differential and shared phosphorylated sites in two comparison groups. Differentially expressed phosphorylated proteins (DEPPs) were mapped onto the protein–protein interaction (PPI) network database of STRING (v.10.5) (http://string-db.org/), and the protein interaction relationship was extracted according to a confidence score > 0.7. Cytoscape software (3.10.0) was used to visualize the PPI network.

## Results

### Identification and quantification of phosphopeptides

**Fig. 1:**
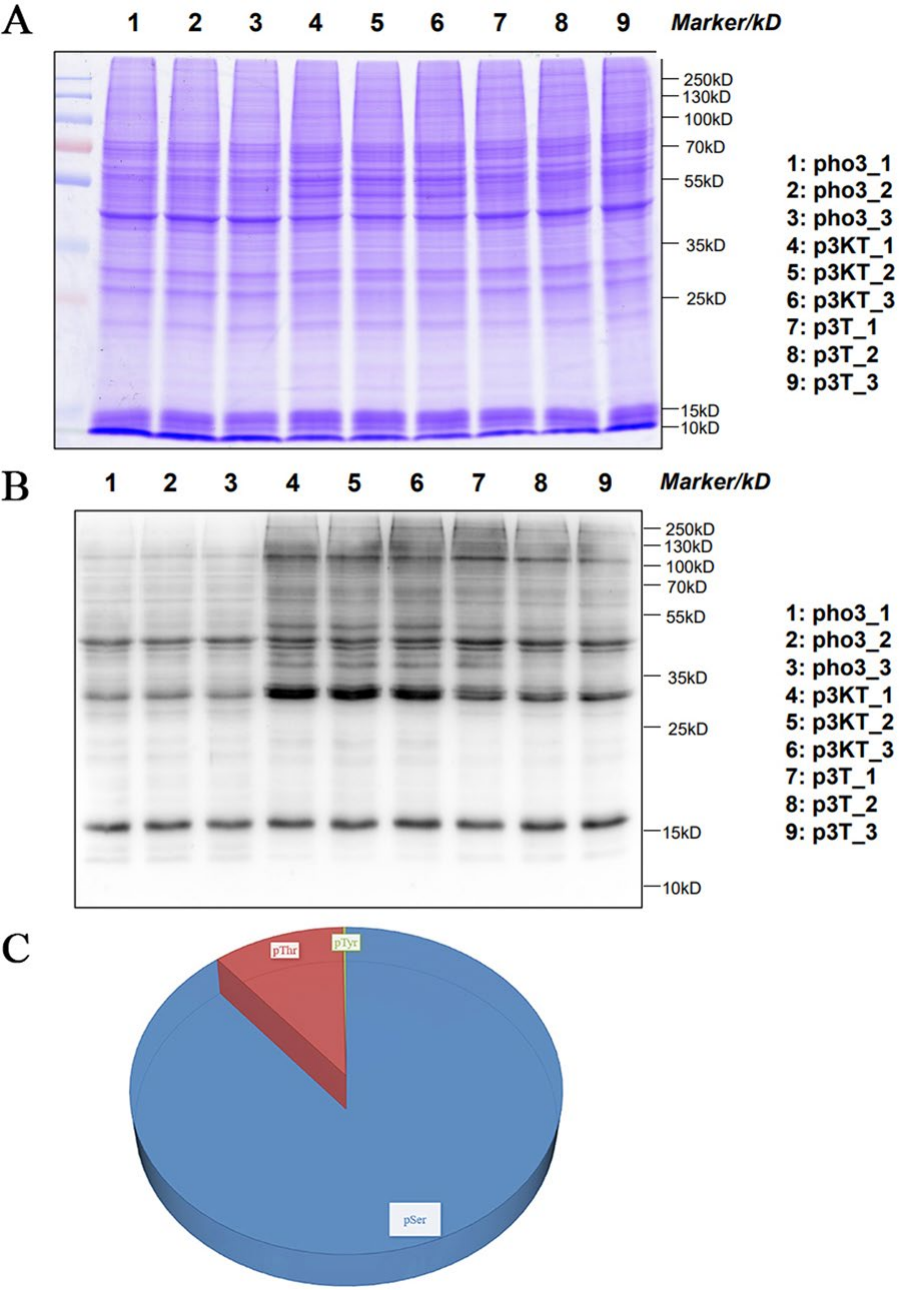
Identification and quantification of phosphopeptides. **A** Results for protein in the sample detected by SDS–PAGE (Coomassie brilliant blue staining). **B** Results for total threonine (Thr) phosphorylation levels detected by western blot; **C** Distribution of phosphorylation on serine (pSer), threonine (pThr), and tyrosine (pTyr) for all phosphorylation sites

The results of total proteins obtained from the three groups (pho3, p3T, and p3KT) analyzed by SDS–PAGE and western blot are shown in Fig. 1. The three biological replicates of each sample had high homogeneity (Fig. 1A), whereas total threonine phosphorylation differed significantly among the three groups (Fig. 1B). Total proteins obtained from the three groups were digested, and phosphopeptides were enriched using the IMAC method. The enriched fractions were analyzed using LC–MS/MS. A total of 127,423, 135,498, and 137,639 spectra were obtained from pho3, p3T, and p3KT, respectively, and the numbers of available and effective spectra were 59,630 (46.80%), 60,354 (44.54%), and 47,312 (34.37%). In addition, 7635, 7706, and 7156 phosphorylated peptides were identified from pho3, p3T, and p3KT, respectively (Table 1).

**Table 1:** Quantification of phosphopeptides

Groups	Total spectra	Matched spectra	Peptides	Modified peptides	Identified proteins	Identified sites
p3KT	137,639	47,312	9143	7156	3009	8732
p3T	135,498	60,354	9784	7706	3229	9383
pho3	127,423	59,630	10,014	7635	3249	9222
Total	400,560	167,296	28,941	22,497	9487	27,337

In order to verify the MS data, we first conducted Pearson’s correlation coefficient (PCC) analysis of these identified peptides and showed that the minimum value of the PCC of three replicates of each sample was 0.98 (Additional file 1: Fig. S1A), which was consistent with SDS–PAGE results. The length of most peptides ranged from 7 to 10 amino acid residues, in line with the attributes of tryptic peptides, indicating the high quality of protein samples (Additional file 1: Fig. S1B). In total, 9222, 9383, and 8732 phosphorylation sites in 3249, 3229, and 3009 proteins were identified from pho3, p3T, and p3KT, respectively (Table 1). Of the 10,029 phosphorylation sites, 8920 (88.95%) were phosphorylated at serine (pSer), 1081 (10.77%) threonine (pThr), and 28 (0.28%) tyrosine (pTyr) (Fig. 1C).

### Differentially expressed phosphoproteins (DEPPs) and clustering analysis

**Fig. 2:**
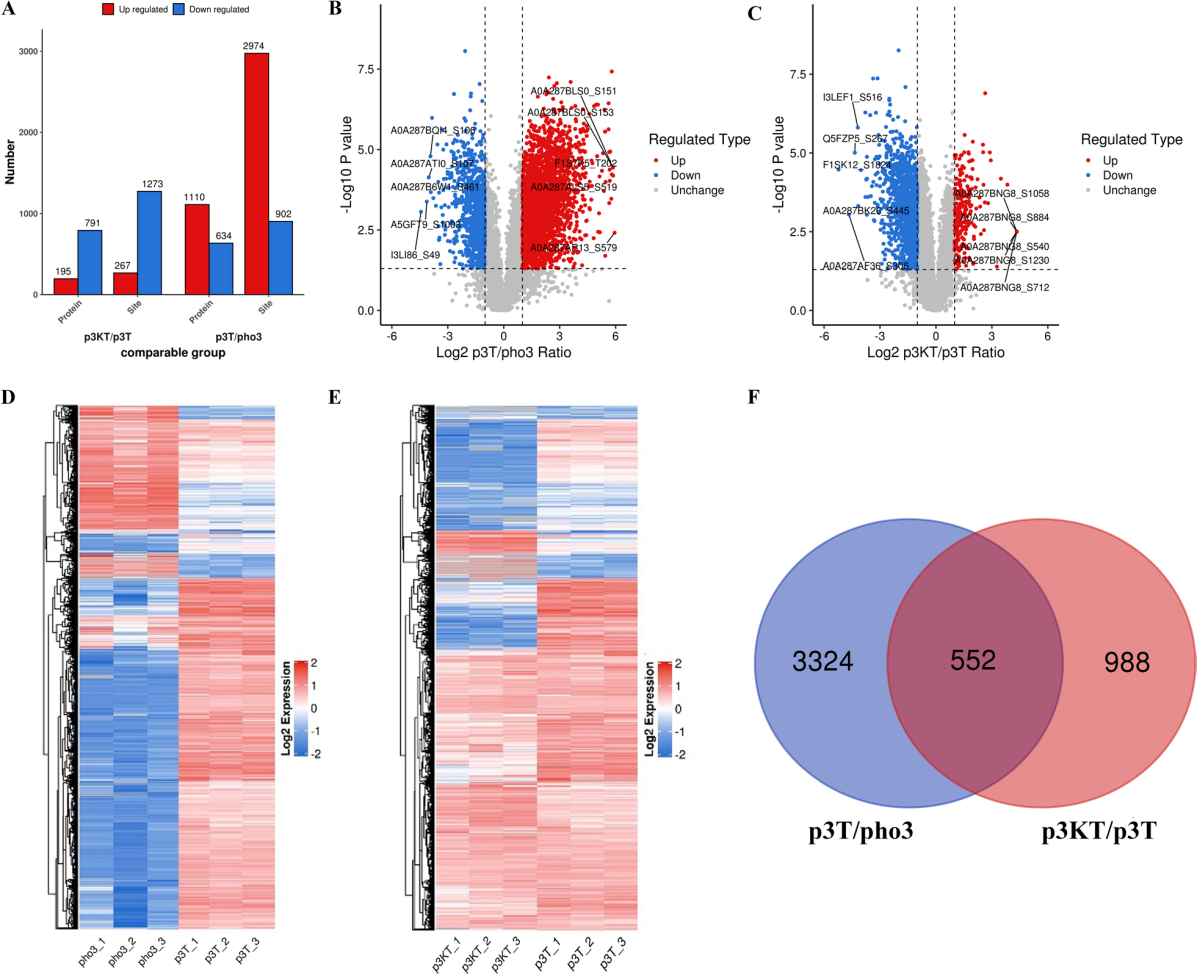
Analysis of differentially phosphorylated proteins. **A** The histogram of differentially expressed phosphorylated proteins (DEPPs). **B**, **C** Volcano plots of differentially phosphorylation sites. The horizontal axis is the log_2_-transformed value of the differential expression change ratio of the comparison group; the vertical axis is the −log_10_-transformed value of the statistical test *t*-test *P* value; where red points indicate significant upregulation of differential modifier loci (*P* < 0.05), blue points indicate significant downregulation (*P* < 0.05), and gray points indicate no significant difference (*P* > 0.05). The top five (absolute log_2_ ratio from largest to smallest) differentially modified loci are also marked on the graph. **D**, **E** Cluster heat map of differentially phosphorylation sites. One differentially modified site per row, one sample per column. Red represents high expression, blue represents low expression. **F** Venn diagram showing the distribution of the differentially phosphorylated sites in two comparison groups

We identified 1647 DEPPs with 3876 differentially phosphorylated sites (DPSs) in p3T when comparing with pho3, and 959 DEPPs with 1540 DPSs in p3KT when comparing with p3T (|log_2_FC|> 1 and *P* < 0.05) (Additional file 2: Table S1 and Additional file 3: Table S2). In the comparison group of p3T/pho3, a total of 2974 DPSs from 1110 proteins were upregulated, and 902 DPSs from 634 proteins were downregulated. In the comparison group of p3KT/p3T, a total of 267 DPSs from 195 proteins were upregulated, and 1273 DPSs from 791 proteins were downregulated (Fig. 2A, B, and C). A heat map of the concatenation of differentially modified loci in all comparison groups was drawn and used to show the relative expression levels of multiple differentially modified sites in different samples, presenting clustering relationships of differentially modified sites (quantitative values were required in at least 2/3 of the total samples) (Figs. 2D and E).

To obtain the DEPPs and DPSs related to A-Raf and regulated during *T. gondii* infection, we compared the DEPPs and DPSs between p3T/pho and p3KT/p3T. DEPPs with the same phosphorylated sites in the comparison of p3T/pho3 versus p3T/p3KT were identified as DEPPs related to A-Raf. Finally, a total of 406 DEPPs with 552 DPSs were identified according to the results of Venn analysis (Fig. 2F, Additional file 4: Table S3). Of these 552 DPSs regulated by A-Raf during *T. gondii* infection, knockout of A-Raf induced dephosphorylation of 80.80% (446) DPSs, but also led to upregulation of phosphorylation levels in 19.20% (106) DPSs.

### Prediction of p-site motifs using Motif-x

**Fig. 3:**
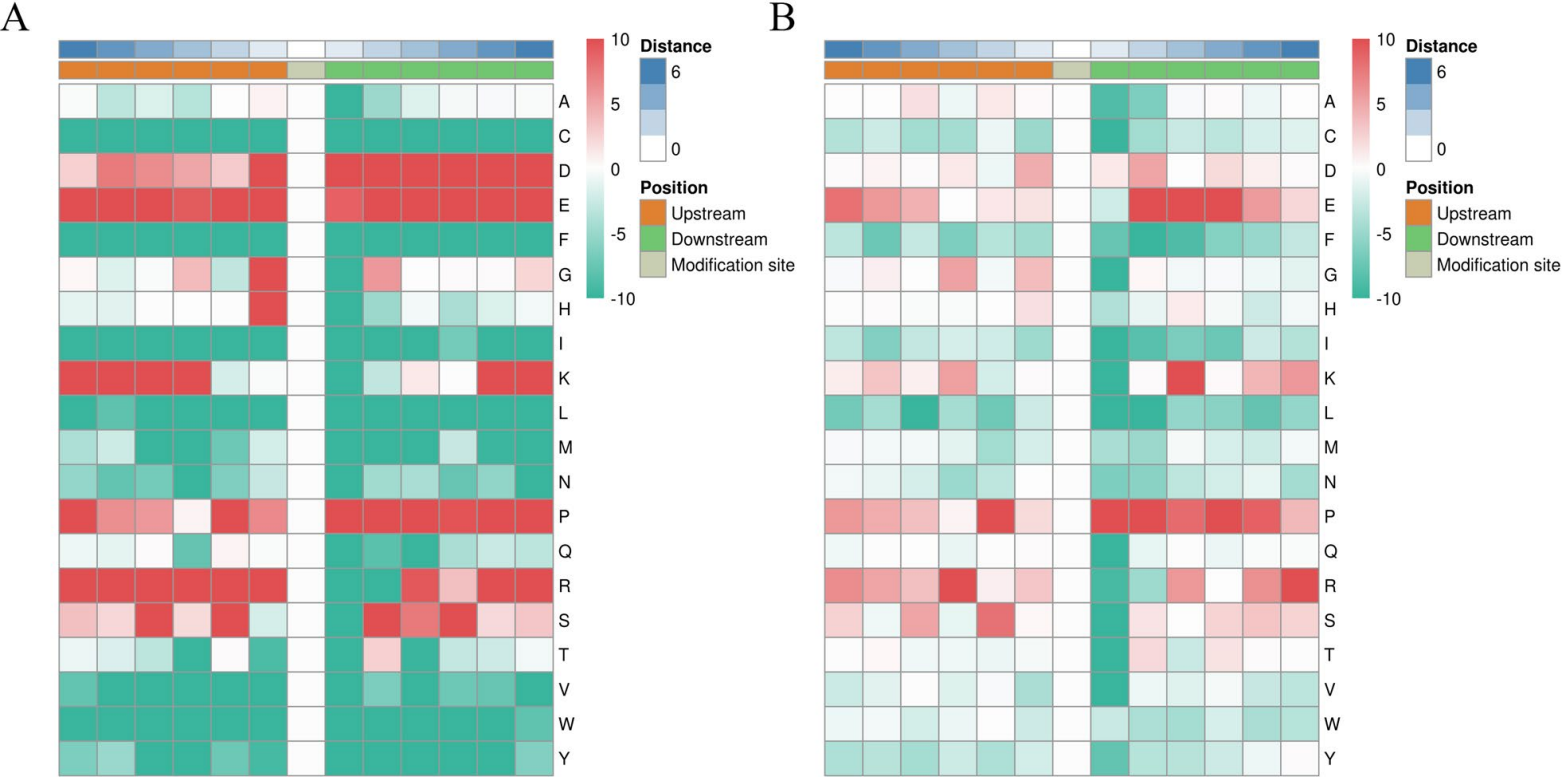
Heat maps of enrichment of six amino acid motifs upstream and downstream of serine **A** and threonine **B** modification sites. All the identified differentially phosphorylated sites (DPSs) data were used for the analyses. The letters on the right of each panel represent amino acids. Red indicates that this amino acid is significantly enriched near the modification site, and green indicates that the secondary amino acid is significantly reduced near the modification site

MoMo software and hierarchical cluster analysis were used to study the phosphosites from six amino acids upstream to six amino acids downstream of the flanking sequences (Fig. 3). All identified DPSs were used for the analyses. The specific amino acid sequence features or motifs around the phosphosites usually determine kinase substrate specificity. The frequency of aspartic (D) and glutamic (E) residues at position −6 to +6 were highest, lysine (K) residues were enriched at −6 to −3 and +5 to +6, proline (P) residues at the positions −6 to −4 and +1 to +6, arginine (R) residues at −6 to −1 and +3 to +6, and serine (S) residues at −6 to −2 and +2 to +6. The frequency of cysteine (C), phenylalanine (F), isoleucine (I), leucine (L), methionine (M), asparagine (N), glutamine (Q), valine (V), tryptophan (W), and tyrosine (Y) were underrepresented in most positions (Fig. 3A and B). In total, 90 motifs were identified. Phosphosites consisted of pSer (85.56%) and pThr (14.44%) residues, and the top ten included KxxxPx_S_Pxxxxx, xxxxPx_S_PxKxxx, xxxxPx_S_PxxxxK, xxxxxx_S_PPxKxx, xxxRSx_S_Pxxxxx, xxxxxx_S_PxxxRR, xxxRxx_S_PxPxxx, xxxxPx_S_Pxxxxx, xxxxxx_S_PxKxxx, and xxxxxx_S_PxRxxx (_S_ indicates the phosphosite, x represents any amino acid residue) (Additional file 5: Table S4).

### Functional enrichment of DEPPs in porcine alveolar macrophages after *T. gondii* infection

To better clarify the potential function of phosphorylation and the biological function of differentially modified proteins in 3D4/21 cells after *T. gondii* infection, functional enrichment analysis of GO terms and KEGG pathways was performed on DEPPs between pho3 versus p3T.

**Fig. 4:**
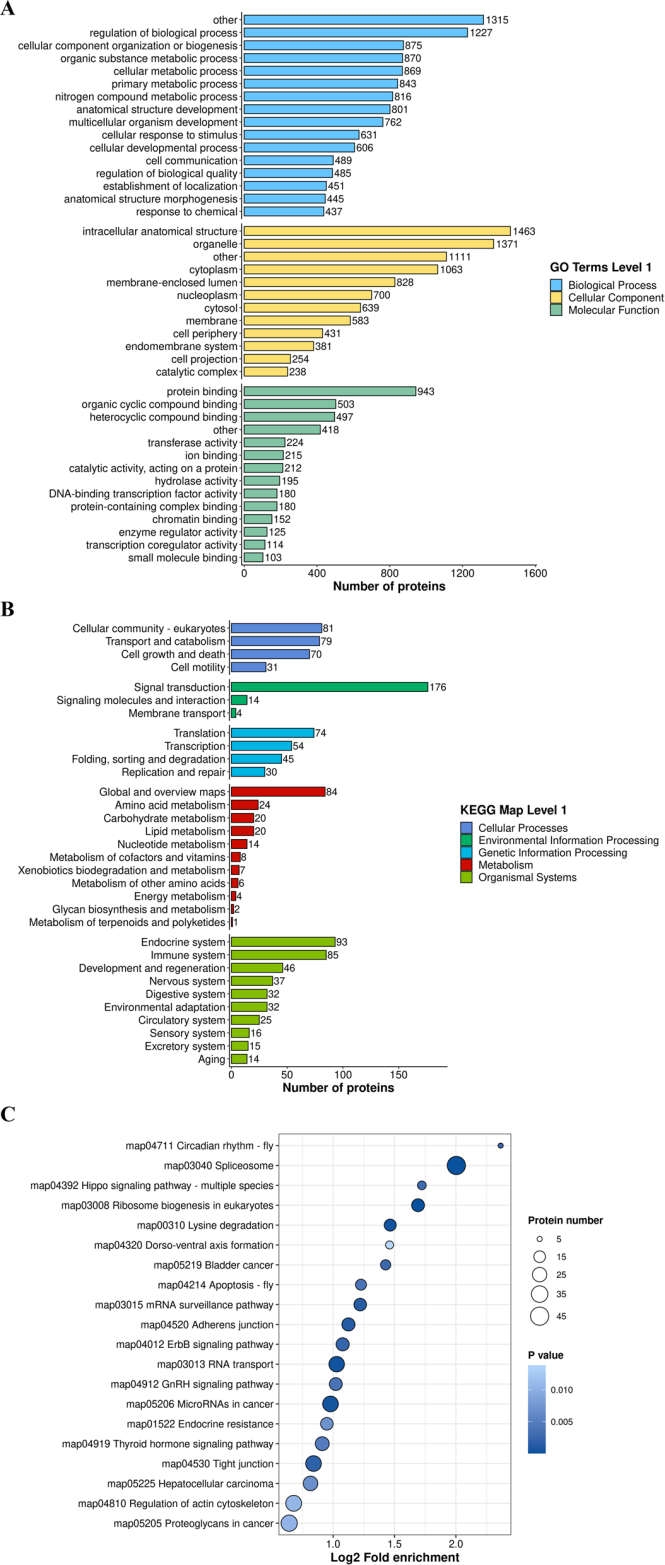
Functional enrichment of DEPPs of p3T/pho3. The *X* axis indicates the number of DEPPs. The Y-axis represents GO terms (**A**) and KEGG pathway maps (**B**). **C** The scatter plots represent KEGG pathway enrichment of the DEPPs of p3T/pho3. KEGG pathway analysis of DEPPs: the vertical axis shows the significantly enriched KEGG pathways and the horizontal axis represents the rich factors corresponding to the pathways. Rich factor refers to the ratio of the number of DEPPs to the number of all phosphoproteins in the pathway. Higher rich factors indicate greater degrees of enrichment. The size and color of the node represent number of phosphoproteins and *P* value of pathways

GO enrichment classification of DEPPs is shown in Fig. 4A and Additional file 6: Table S5. Of 1647 DEPPs, 1568 were categorized into 42 GO terms, 1501 were categorized into BP, 1514 into CC, and 1338 into MF. Of the 42 GO terms, 16 terms were assigned to BP, 12 terms to CC, and 14 terms to MF. For the BP category, the significantly enriched DEPPs were mainly involved in regulation of biological process (1227 proteins), cellular component organization or biogenesis (875 proteins), and organic substance metabolic process (870 proteins). In the CC category, significantly enriched DEPPs were mainly involved in intracellular anatomical structure (1643 proteins), organelle (1371 proteins), and cytoplasm (1063 proteins). Regarding the MF category, proteins were mainly enriched in binding (947 proteins), organic cyclic compound protein binding (503 proteins), and heterocyclic compound binding (497 proteins).

KEGG pathway annotations of DEPPs are shown in Fig. 4B. KEGG enrichment identified five functional categories, including cellular processes (CP), environmental information processing (EIP), genetic information process (GIP), metabolism, and organismal systems (GS). In the CP category, 81, 79, and 70 DEPPs were enriched in cellular community eukaryotes, transport and catabolism, and growth and death, respectively. In the EIP and metabolism category, the greatest number of DEPPs were enriched in translation (74 proteins), signal transduction (176 proteins), and global and overview maps (84 proteins). In the GS category, 93 and 85 DEPPs were enriched in the endocrine and immune systems, respectively (Additional file 7: Table S6).

KEGG pathway enrichment analysis of DEPPs is shown in Fig. 4C. A total of 391 DEPPs were mapped to 35 pathways (Additional file 8: Table S7), of which spliceosome (45 proteins), proteoglycans in cancer (34 proteins), regulation of actin cytoskeleton (32 proteins), microRNAs in cancer (31 proteins), tight junction (31 proteins), and RNA transport pathway (30 proteins) were the top six. In addition, Notch signaling (9 proteins), apoptosis-fly (13 proteins), hippo signaling pathway-multiple species (8 proteins), hippo signaling pathway-fly (13 proteins), and the ErbB signaling pathway (20 proteins) were predicted to participate in apoptosis, and the ErbB signaling pathway was ranked in the top 20 (Additional file 8: Table S7). Mitogen-activated protein kinase 1 (MAPK1), rapidly accelerated fibrosarcoma 1 (Raf1), son of sevenless 1 (SOS1), B-rapidly accelerated fibrosarcoma (B-Raf), epidermal growth factor receptor (EGFR), A-Raf, BCL2 associated agonist of cell death (Bad), myelocytomatosis oncogene (Myc), Jun proto-oncogene (Jun), and myeloid cell leukemia 1 (Mcl1) were mapped to pathways related to apoptosis or cancers (Additional file 9: Table S8).

### Functional enrichment of DEPPs indirectly or directly related to A-Raf

**Fig. 5:**
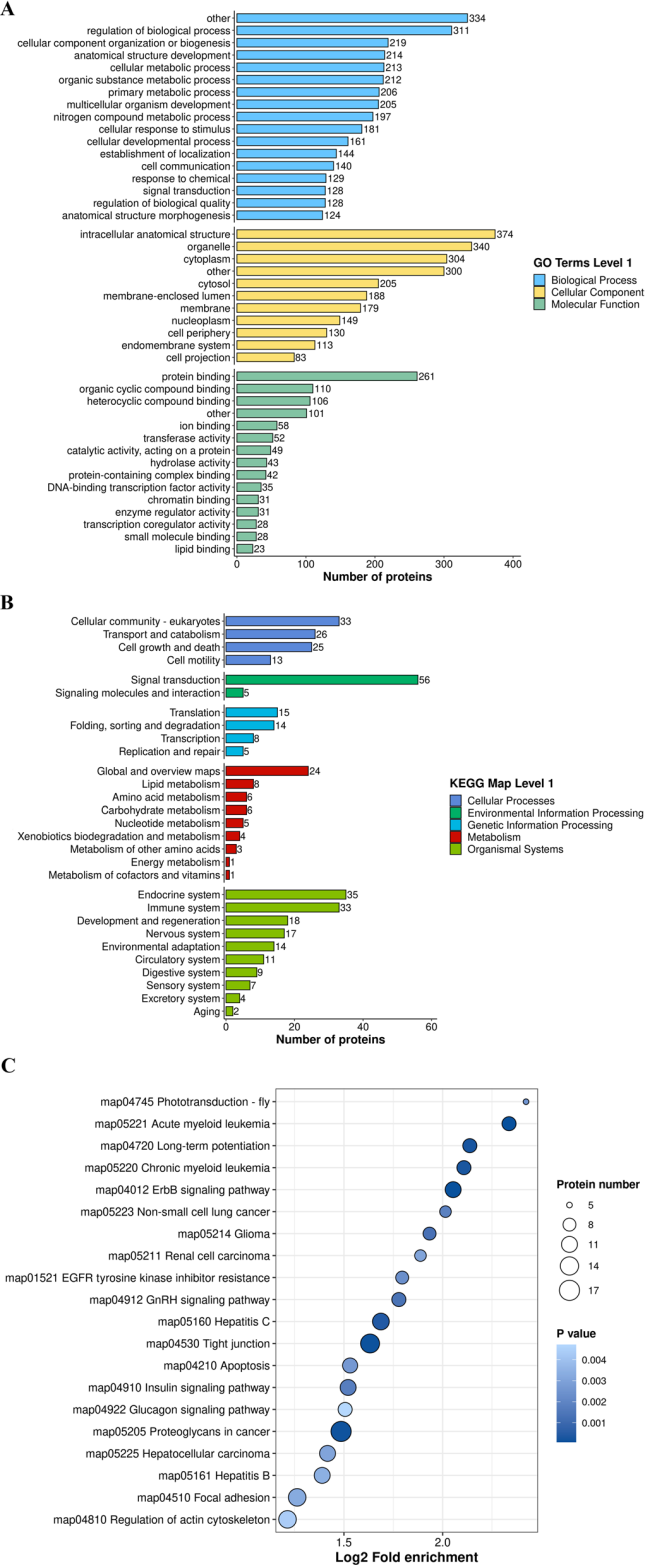
Functional enrichment of DEPPs related to A-Raf and regulated during *T. gondii* infection. DEPPs data set used for the functional analyses was the DEPPs with the same phosphorylated sites in the comparison of p3T/pho3 versus p3T/p3KT. The *X* axis indicates the number of DEPPs. The *Y* axis represents GO terms (**A**) and KEGG pathway maps (**B**). **C** KEGG pathway enrichment of the DEPPs related to A-Raf

To explore the potential roles of A-Raf in host cells during *T. gondii* infection, DEPPs regulated by A-Raf underwent GO functional enrichment and KEGG pathway analysis. DEPPs related to A-Raf and regulated during *T. gondii* infection were identified by Venn analysis. As shown in Fig. 5A and Additional file 6: Table S5, of the 406 (total) DEPPs, 394 DEPPs were categorized into 43 GO terms, 373 DEPPs were categorized as BP, 384 DEPPs as CC, and 338 as MF. Of 43 GO terms, 17 terms were assigned to BP, 11 terms to CC, and 15 terms to MF. One GO term (catalytic complex) in the CC category was missing, whereas two terms (signal transduction in the BP category and lipid binding in the MF category) were added when comparing the GO enrichment results of DEPPs between pho3 vs. p3T (Additional file 6: Table S5).

The results of KEGG pathway annotations of DEPPs were similar to those of DEPPs between pho3 and p3T (Fig. 5B). However, three pathway maps were missing, including membrane transport under EIP, glycan biosynthesis and metabolism under metabolism, and metabolism of terpenoids and polyketides under metabolism, when comparing the KEGG pathway annotation results of DEPPs between pho3 and p3T (Additional file 7: Table S6).

KEGG pathway enrichment results of DEPPs related to A-Raf are shown in Fig. 5C. Of 406 DEPPs, 108 were mapped to 50 pathways, of which proteoglycans in cancer (17 proteins), tight junction (15 proteins), endocytosis (14 proteins), regulation of actin cytoskeleton (13 proteins), and focal adhesion (13 proteins) were the top five (Additional file 8: Table S7). A total of 14 pathways, including notch signaling, apoptosis-fly, and hippo signaling pathway-multiple species pathway, were missing, whereas 29 pathways, including apoptosis (10 proteins) pathways, were added when comparing KEGG pathway enrichment result of DEPPs between pho3 and p3T (Additional file 8: Table S7). Raf1, B-Raf, EGFR, SOS1, Bad, Myc, Jun, and Mcl1 were mapped to pathways related to apoptosis or cancers (Additional file 9: Table S8).

### Protein–protein interaction (PPI) networks of phosphorylated proteins related to A-Raf

**Fig. 6:**
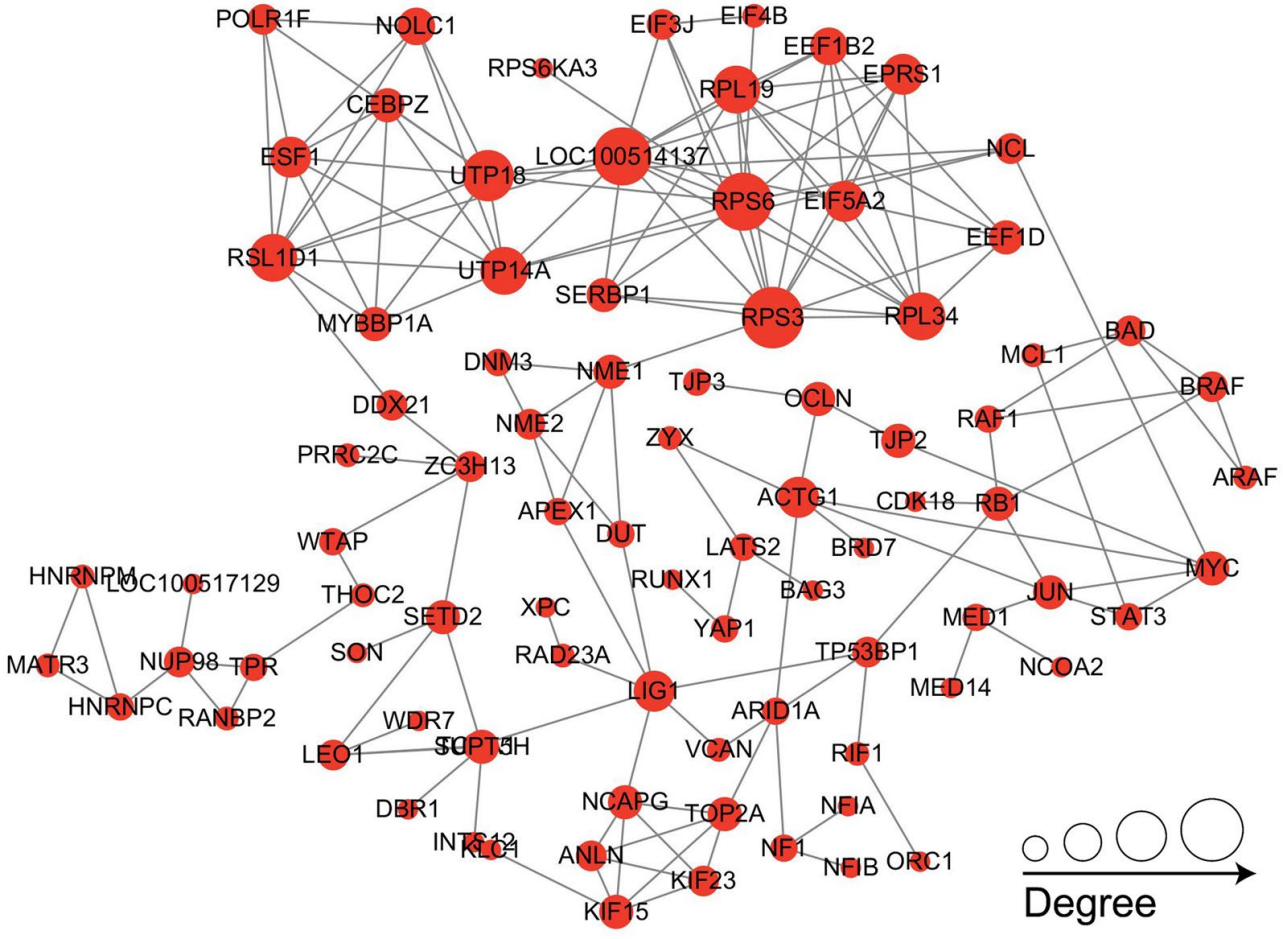
Protein–protein interaction (PPI) networks of the DEPPs related to A-Raf. Cytoscape software and String database were used to construct the PPI networks. Nodes represent differentially expressed proteins. Size of the node represents number of the differential proteins and their interacting proteins

PPI networks (combined score > 0.7) were constructed to understand the regulatory mechanisms of PTMs and to identify the relevant functional clusters of the 406 DEPPs related to A-Raf in host cells during *T. gondii* infection. As shown in Fig. 6, a total of 86 nodes were identified in the PPI network. Significant hub proteins identified included many proteins related to apoptosis, such as Jun, Bad, Mcl1, mediator complex subunit 1 (Med1), and Myc.

### Potential phosphorylated proteins and sites involved in host-cell apoptosis

**Fig. 7:**
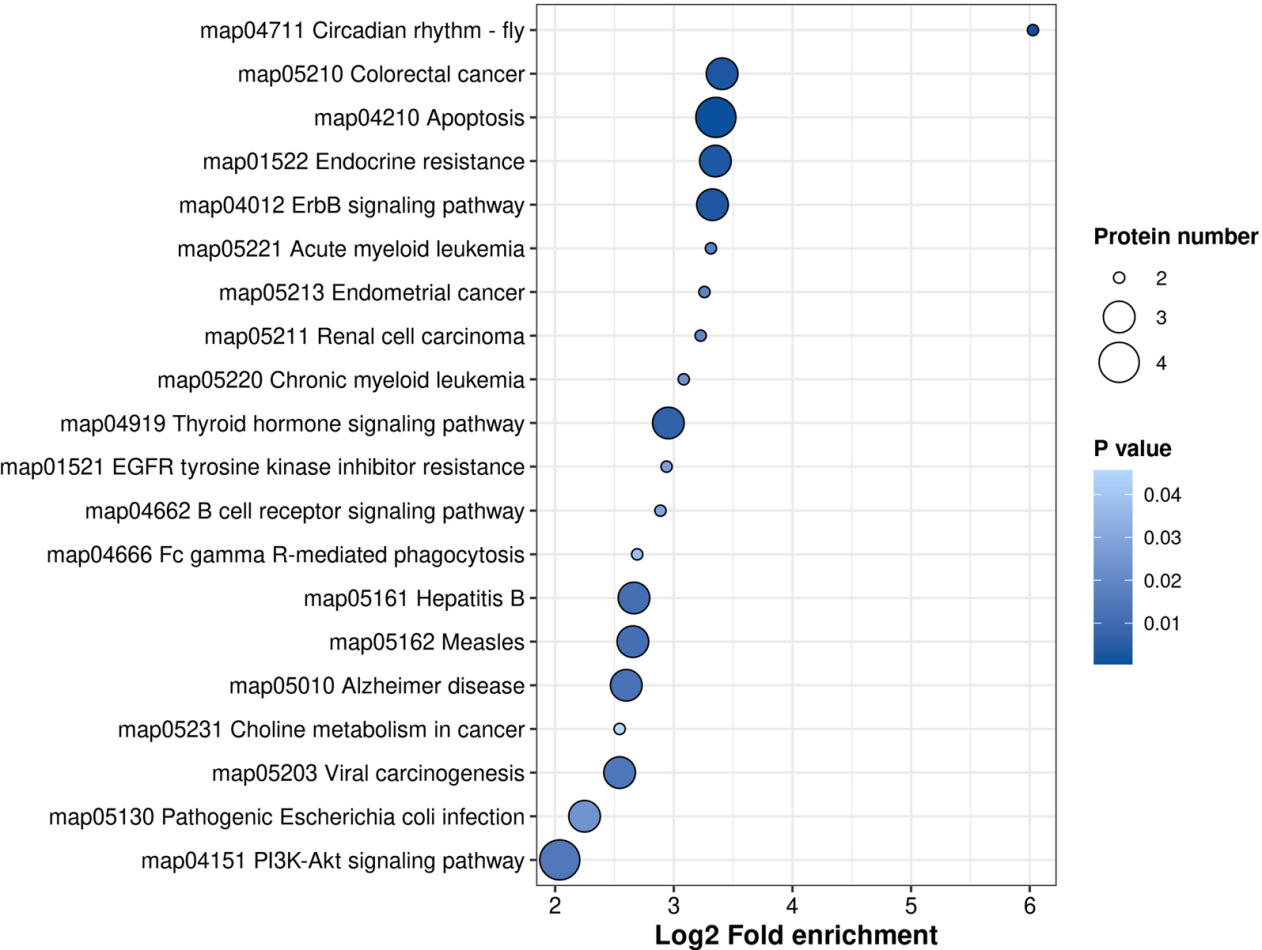
KEGG pathway enrichment of the DEPPs related to A-Raf associated with apoptosis

In a previous study, we found that A-Raf knockout could reduce the apoptosis rate of 3D4/21-Δ*Araf* cells during *T. gondii* infection (Su et al., 2023). From the analysis above, we also found many DEPPs that may be associated with apoptosis. Here, to identify the putative phosphorylated proteins and sites possibly involved in host-cell apoptosis in our dataset, the potential function of each DEPP related to A-Raf was analyzed using GO annotation or KEGG pathway enrichment, and the DEPPs potentially involved in apoptosis were identified as apoptosis-associated proteins. Thus, 40 of 406 DEPPs were screened out (Table 2). The 40 DEPPS were then analyzed using KEGG pathway enrichment, in which 16 of 40 DEPPs were mapped to 20 pathways, including the PI3K-Akt signaling pathway, apoptosis, and ErbB signaling pathway (Fig. 7, Additional file 10: Table S9). These pathways may be related to apoptosis of host cells during *T. gondii* infection. Further analysis revealed that Jun, Myc, Mcl1, Med1, and Bad participated in multiple pathways (Additional file 9: Table S8), and that the phosphorylation levels of Med1 at serine 1418, Jun at serine 73, Myc at serine 154, Mcl1 at serine 65, and Bad at serine 115 were upregulated in p3T but downregulated in p3KT (Table 2). Meanwhile, the results of PPI analysis showed that these five proteins interacted with each other in the network of 406 DEPPs related to A-Raf (Fig. 6). Altogether, these results suggest that A-Raf may regulate phosphorylation of these proteins and sites to modulate *T. gondii*-induced apoptosis in macrophages.

**Table 2:** DPSs and DEPPs related with A-Raf associated with apoptosis

Protein accession	Position	Amino acid	Gene name	Change trend in DEPPs of P3T/pho3	Change trend in DEPPs related with A-Raf	Protein description
F1S1C4	260	T	LOC110260307	Down	Up	Fc fragment of IgG, low affinity IIb, receptor (CD32)
F1REZ3	281	S	VCAN	Down	Up	Versican
A0A287ATI0	107	S	ARHGEF2	Down	Up	Rho guanine nucleotide exchange factor 2
A0A286ZJK8	176	S	UNC13B	Down	Up	Unc-13 homolog B
A0A287B984	201	S	BNIP2	Down	Up	BCL2 interacting protein 2
A0A287B833	615	S	ATXN2	Up	Down	Ataxin 2
P60588	272	T	PLPP1	Up	Down	Phospholipid phosphatase 1
A0A5G2QE26	227	S	MECP2	Up	Down	Methyl-CpG-binding protein 2
F2Z5Q6	235	S	RPS6	Up	Down	40S ribosomal protein S6
A0A287APV2	856	S	–	Up	Down	Myosin motor domain-containing protein
F1S0L8	308	S	MTDH	Up	Down	Metadherin
A0A287BLA6	1672	S	MAP2	Up	Down	Microtubule-associated protein
A0A5K1UB22	965	S	HIPK1	Up	Down	Homeodomain interacting protein kinase 1
A0A481ARY0	393	S	RSL1D1	Up	Down	Ribosomal L1 domain containing 1
F1SK12	2277	S	MAP1B	Up	Down	Microtubule associated protein 1B
A0A287BBE4	1497	T	MYBBP1A	Up	Down	MYB binding protein 1a
F1RR06	83	S	NMT1	Up	Down	Glycylpeptide N-tetradecanoyltransferase
A0A287BLA6	1849	S	MAP2	Up	Down	Microtubule-associated protein
A0A287AI98	506	S	ATXN2L	Up	Down	Ataxin 2 like
F1S415	210	S	BAG3	Up	Down	BAG cochaperone 3
F1RPI9	25	S	ATRX	Up	Down	DNA helicase
F1S827	234	S	SERBP1	Up	Down	Plasminogen activator inhibitor 1 RNA-binding protein isoform 1
K9IVT3	691	S	RBBP6	Up	Down	E3 ubiquitin-protein ligase RBBP6
F1RPI9	746	S	ATRX	Up	Down	DNA helicase
F1RPI9	701	S	ATRX	Up	Down	DNA helicase
F1SMV6	28	S	NCL	Up	Down	Nucleolin
F1RPI9	675	S	ATRX	Up	Down	DNA helicase
A0A287AZL3	59	S	KRT5	Up	Down	Keratin 5
F1RPI9	673	S	ATRX	Up	Down	DNA helicase
A0A287B5Q4	457	S	RTN4	Up	Down	Reticulon
P56432	73	S	JUN	Up	Down	Transcription factor Jun
F1SP80	204	S	PLAA	Up	Down	Caspase activity and apoptosis inhibitor 1 isoform 1
A0A287B5Q4	453	S	RTN4	Up	Down	Reticulon
F1RPI9	848	S	ATRX	Up	Down	DNA helicase
F1RPI9	34	S	ATRX	Up	Down	DNA helicase
F1RPI9	1060	S	ATRX	Up	Down	DNA helicase
A0A287A7H5	541	S	WTAP	Up	Down	Pre-mRNA-splicing regulator WTAP
F1S415	327	S	BAG3	Up	Down	BAG cochaperone 3
A0A287AEF3	115	S	BAD	Up	Down	BCL2 associated agonist of cell death
A0A287BKE2	333	S	NDRG1	Up	Down	Protein NDRG1
P60588	265	T	PLPP1	Up	Down	Phospholipid phosphatase 1
A0A5G2QA41	151	S	RTN4	Up	Down	Reticulon
K9IWB2	65	S	MCL1	Up	Down	Induced myeloid leukemia cell differentiation protein Mcl-1
F1RPI9	592	S	ATRX	Up	Down	DNA helicase
F1RPI9	596	S	ATRX	Up	Down	DNA helicase
A0A287AUM0	1633	S	PTPN13	Up	Down	Tyrosine-protein phosphatase non-receptor type 13
F1RPI9	849	S	ATRX	Up	Down	DNA helicase
A0A287BBE4	1503	T	MYBBP1A	Up	Down	MYB binding protein 1a
F1RWL2	1418	S	MED1	Up	Down	Mediator of RNA polymerase II transcription subunit 1
F1RR06	47	S	NMT1	Up	Down	Glycylpeptide N-tetradecanoyltransferase
A0A5G2QA41	147	S	RTN4	Up	Down	Reticulon
A0A5G2QN58	154	S	MYC	Up	Down	Myc proto-oncogene protein
A0A287B464	2743	S	MYCBP2	Up	Down	RCR-type E3 ubiquitin transferase
F1RRB5	743	S	JADE1	Up	Down	Jade family PHD finger 1
A0A287BF97	523	S	EEF1D	Up	Down	Eukaryotic translation elongation factor 1 delta
A0A5G2R0A4	230	S	LOC100517129	Up	Down	HSR domain-containing protein
F1S642	659	S	TP53BP2	Up	Down	Tumor protein p53 binding protein 2

## Discussion

PTMs on proteins transmit signals between parasites and their hosts, playing a key role during the process of parasite development [28, 29]. In Asia, including China, the predominant *T. gondii* genotype is Chinese 1 (ToxoDB#9), which accounts for almost 78% of all strains of *T. gondii* in China and has similar virulence to the type 1 RH strain [23, 30, 31]. Protein phosphorylation plays a key role in host cell–*T. gondii* interactions [32]. However, the phosphoproteomic profile of porcine alveolar macrophages during *T. gondii* Chinese 1 genotype infection has not been thoroughly described. In addition, the role of A-Raf in host cell–*T. gondii* interactions has not yet been reported. In the present study, we initially assessed phosphoproteomic data from pig macrophages (3D4/21 cells) using IMAC in combination with LC–MS/MS during *T. gondii* infection. Functional analysis of these DEPPs in the macrophages during parasite infection showed that *T. gondii* may modulate host cell processes through phosphorylation, such as regulating biological processes, cell growth and death, membrane transport, signal transduction, and immune responses, inducing apoptosis, blocking the synthesis of some inflammatory factors, utilizing the membrane, and mediating metabolism to support the proliferation and release of parasites. Recent research analyzed changes in the levels of phosphorylation modifications of 3D4/21 cells at 24 h after *T. gondii* RH strain infection. The author identified 8063 DPSs and 2607 DEPPs. Functional analysis revealed that these DEPPs were involved in glycogenesis, transcription activation, and apoptosis of host cells [33]. Other research compared the phosphoproteome of HFF cells 30 min and 28 h after *T. gondii* RH strain infection. The authors identified 58 upregulated and 23 downregulated DEPPS in the infection group compared to the non-infection group at 28 h PI. Functional analysis of these DEPPs revealed that apoptosis was a more important biological process in host cells at 28 h PI than at 30 min PI [34]. Compared with previous studies [33, 34], our results identified different numbers of DEPPs and DPSs, which may be due to differences in the *T. gondii* strain or the host cells.

A-Raf is considered a crucial signaling hub controlling cellular processes such as proliferation, apoptosis, and glucose metabolism [35]. A-Raf has also been demonstrated to play an important role in tumorigenesis [17, 36]. A-Raf dysregulation is closely associated with the initiation and progression of neoplastic diseases [17, 36]. In cancer cells, A-Raf kinase can inhibit apoptosis by binding to the proapoptotic mammalian sterile 20-like kinase (MST2) protein and inhibiting its kinase activity [17]. A-Raf can block the Hippo signaling pathway mediated by MST2 phosphorylation [19]. To better explore the potential impact of A-Raf on downstream pathways in this study, we have analyzed all DEPPs and DPSs that were regulated by A-Raf. In p3T/pho3 comparison, the DPSs and DEPPs were expected to respond to *T. gondii* infection, whereas in p3T/p3KT comparison, the DPSs and DEPPs were expected to respond to A-Raf knockout in host cells. The shared DPSs and DEPPs in two comparison groups (p3T/pho3, p3T/p3KT) were expected to respond to A-Raf knockout in the presence of *T. gondii* infection, which were directly or indirectly related to A-Raf in the host cells with *T. gondii* infection. Ultimately, we identified 406 shared DEPPs and 552 DPSs related to A-Raf and *T. gondii* infection by comparing the DEPPs and DPSs between p3T/pho and p3KT/p3T. Of the 552 DPSs, 446 (80.80%) DPSs regulated by A-Raf were dephosphorylated, suggesting that A-Raf, as a serine/threonine protein kinase, may act as a “switch” for the biological functions involved in these proteins. Nevertheless, A-Raf knockout was also found to significantly increase phosphorylation levels of some proteins (19.20%). This reverse activation effect has been demonstrated in other studies, for example, a previous study revealed that knockdown of AKT2 (AKT serine/threonine kinase 2) could significantly increase MEK1/2 and p70S6K1 (p70 KDa ribosomal protein S6 kinase 1) phosphorylation [37]. Additionally, we could not determine whether different abundances of phosphoproteins were due to different expression levels of the protein or different phosphorylation levels. It was possible that the non-differentially expressed proteins showed dephosphorylation and these proteins or peptides might not be the direct targets of A-Raf. So, the available data could not affirm that these common DPSs must be the targets of A-Raf or directly related to A-Raf. But these findings indicated that A-Raf regulated the phosphorylation and dephosphorylation of these proteins, either directly or indirectly, suggesting that it is essential for the regulation and control of functional activation of these proteins in *T. gondii*-infected cells. Functional analysis revealed 40 DEPPs corresponding to 57 DPSs involved in the apoptosis of 3D4/21 cells during *T. gondii* infection. Of these 40 DEPPs, 16 were mapped to 20 pathways, including PI3K-Akt signaling pathway, apoptosis, ErbB signaling pathway, and three pathways related to cancer, but not the Hippo signaling pathway. In addition, the phosphorylation levels of MST2 showed no difference between the p3T and pho groups, whereas phosphorylation of MST2 in the p3KT group was significantly reduced when compared to the p3T group. Therefore, we suggest that A-Raf does not utilize the MST2 phosphorylation-mediated Hippo signaling pathway to modulate apoptosis of macrophages induced by *T. gondii* infection. Meanwhile, previous studies and the PPI network of 406 DEPPs related to A-Raf have revealed that Jun [38], Myc [39–41], Mcl1 [42], Med1 [43], and Bad [44] are able to interact with one another and are associated with apoptosis.

Phosphorylation of Jun is associated with the activation of JNK. It has been reported that phosphorylation of Jun at S73 can protect Jun from degradation by ubiquitination and promote its transcriptional effects, resulting in high expression, while highly expressed Jun can promote apoptosis [38]. In this study, Jun was phosphorylated at S73 after *T. gondii* infection and dephosphorylated after A-Raf knockout in 3D4/21 cells. In our previous study, we found that *T. gondii* infection would induce apoptosis in host cells, but the level of the apoptosis would decrease after A-Raf was knocked out of host cells [22]. We suggested that Jun phosphorylated at S73 might promote apoptosis during the regulation of apoptosis by A-Raf. Myc has been reported to play a key role in tumorigenesis and therapeutic resistance [39]. Myc phosphorylated at different sites will have different functions [45]. Mcl1 belongs to the Bcl2 family and exerts an anti-apoptotic effect in cells. The Mcl1 phosphorylation at difference sites could influence Mcl1 stability and Mcl1 stability could influence its regulation of apoptosis [42, 46–51]. The stability of Myc and Mcl1 both can be altered by their phosphorylation, while the stability of Myc and Mcl1 could determine their regulation of apoptosis. In this study, Myc and Mcl1 were phosphorylated after *T. gondii* infection and dephosphorylated after A-Raf knockout in 3D4/21 cells at S154 and S65, respectively. We hypothesized that Myc and Mcl1 phosphorylated at S154 and S65, respectively, might affect their stability, making them more susceptible to degradation and then promoting apoptosis. Med1 is involved in tumorigenesis and cancer progression through its pleotropic functions in various cellular processes [52, 53]. Phosphorylation of Med1 at S671 [54] and at T1457 [55] also have been shown to be involved in functions related to apoptosis or proliferation. Med1 has also been shown to regulate the Jun amino-terminal kinase (JNK)/c-Jun pathway [43]. Bad is a multifunctional member of the Bcl-2 family with both apoptosis-regulating functions and nonapoptotic functions. Its apoptotic and metabolic functions are determined by its phosphorylation status [56, 57]. Both phosphorylation and dephosphorylation of BAD could promote apoptosis [44, 56–59]. Phosphorylation of Med1 and BAD could be involved in the regulation of apoptosis. Combining this study and previous studies, we hypothesized that phosphorylation of Med1 at serine1418 and Bad at serine115 might promote apoptosis. We speculated that there may be a Jun/Myc/Mcl1/Med1/Bad axis involved in the regulation of apoptosis by A-Raf in host cells during *T. gondii* infection. These conclusions were speculated in the context of this study and previous studies, we will further confirm these conclusions with more studies, such as gene knockout technology or histological sequencing. Taken together, phosphorylation of Med1 at serine1418, Jun at serine 73, Myc at serine 154, Mcl1 at serine 65, and Bad at serine115 might modulate macrophage apoptosis induced by *T. gondii* infection.

In a previous study, we demonstrated that miR-185/A-Raf can regulate apoptosis induced by *T. gondii* infection [22]. Apoptosis is crucial for *T. gondii* to successfully infect host cells. The results of this study further elucidated the potential roles of A-Raf in host cells with *T. gondii* infection, which may provide new ideas in the search for toxoplasmosis prevention and control strategies.

## Conclusions

In summary, our research is the first to examine the global phosphoproteome of porcine alveolar macrophages (3D4/21 cells) with *T. gondii* infection. We identified 552 DPSs corresponding to 406 DEPPs that may be directly or indirectly controlled by A-Raf. These DEPs were significantly enriched in several pathways related to the apoptosis of 3D4/21 cells during *T. gondii* infection. The phosphorylation and dephosphorylation of Med1 at serine1418, Jun at serine 73, Myc at serine 154, Mcl1 at serine 65, and Bad at serine115 may modulate *T. gondii*-induced apoptosis in macrophages. The function of these phosphorylated sites should be confirmed in subsequent studies.

### Supplementary Information

**Additional file 1: Figure S1.** Identification and quantification of phosphopeptides. (A) Pearson’s Correlation Coefficient of protein quantitation; (B) The length distribution of the peptides.

**Additional file 2: Table S1.** Details of DPSs and DEPPs of p3T/pho3.

**Additional file 3:  Table S2.** Details of DPSs and DEPPs of p3KT/p3T.

**Additional file 4: Table S3.** DPSs and DEPPs related to A-Raf.

**Additional file 5: Table S4.** Details of prediction of p-site motifs using Motif-x.

**Additional file 6: Table S5.** GO annotation results of DEPPs of p3T/pho3 or related to A-Raf.

**Additional file 7: Table S6.** KEGG pathway annotation results of DEPPs of p3T/pho3 or related to A-Raf.

**Additional file 8: Table S7.** KEGG pathway enrichment results of DEPPs of p3T/pho3 or related to A-Raf.

**Additional file 9: Table S8.** KEGG pathway enrichment results of MAPK1, Raf1, B-Raf, A-Raf, EGFR, SOS1, Bad, Myc, Jun and Mcl1.

**Additional file 10: Table S9.** KEGG pathway enrichment results of DEPPs related to A-Raf associated with apoptosis.

## Data Availability

The datasets supporting the findings of this article are included within the article. All the mass spectrometry data have been submitted to the ProteomeXchange Consortium with identifier PXD044725 (username: reviewer_pxd044725@ebi.ac.uk; Password: SD2X0Yny).
