# Supplementary figures and images for: Phosphoproteomic analysis reveals changes in A-Raf-related protein phosphorylation in response to Toxoplasma gondii infection in porcine macrophages
Source: Parasit Vectors. 2024 Apr 20;17:191. doi: 10.1186/s13071-024-06273-x (PMC11031963; doi:10.1186/s13071-024-06273-x)

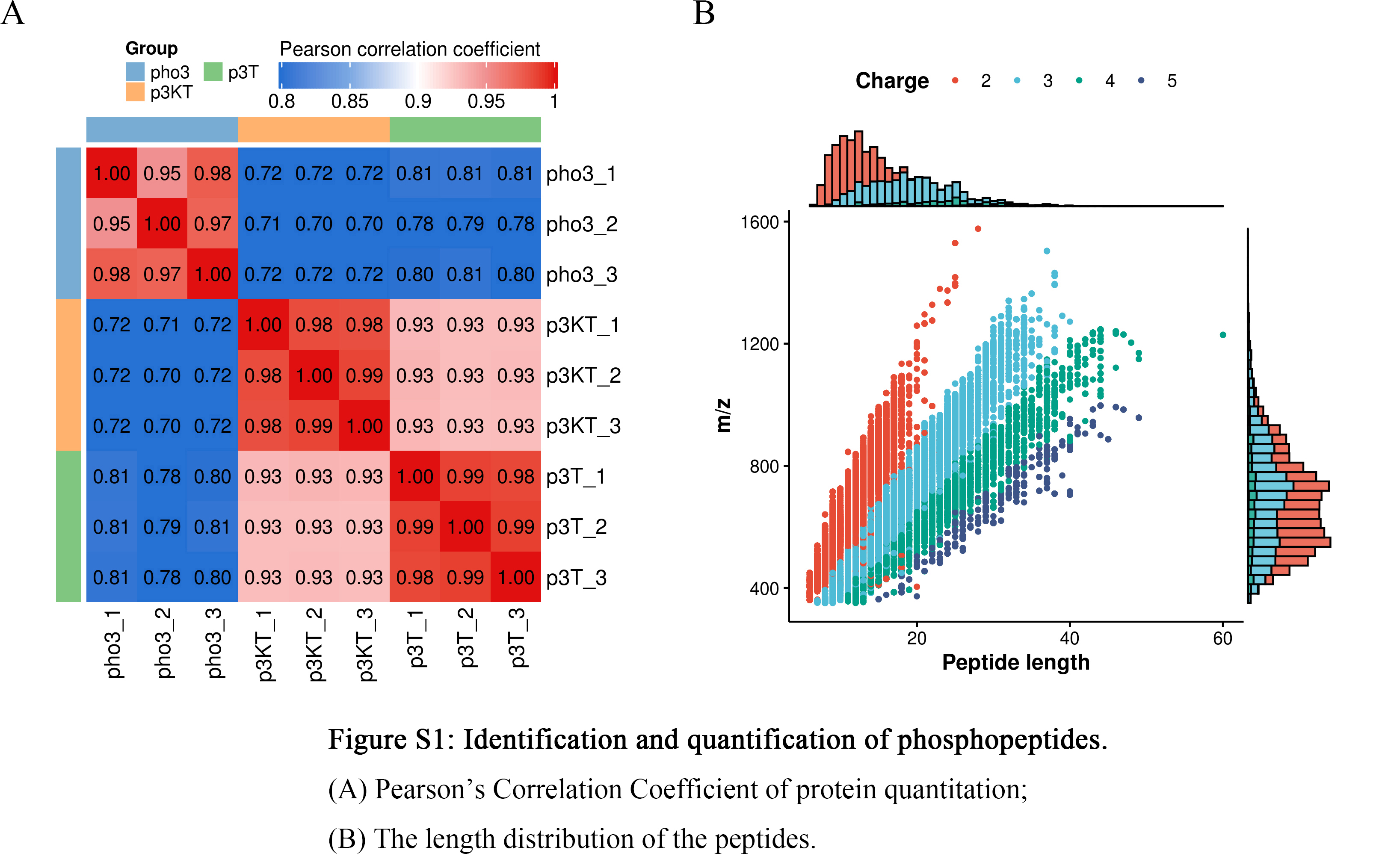

Supplement: Supplementary file 1 — Additional file 1: Figure S1. Identification and quantification of phosphopeptides. (A) Pearson’s Correlation Coefficient of protein quantitation; (B) The length distribution of the peptides. [file 13071_2024_6273_MOESM1_ESM.jpg]
